# An immune-related gene prognostic index for predicting prognosis in patients with colorectal cancer

**DOI:** 10.3389/fimmu.2023.1156488

**Published:** 2023-07-06

**Authors:** Chao Li, Ulrich Wirth, Josefine Schardey, Viktor V. Ehrlich-Treuenstätt, Alexandr V. Bazhin, Jens Werner, Florian Kühn

**Affiliations:** ^1^ Department of General, Visceral, and Transplant Surgery, Ludwig-Maximilians-University Munich, Munich, Germany; ^2^ German Cancer Consortium (DKTK), Munich, Germany; ^3^ Bavarian Cancer Research Center (BZKF), Munich, Germany

**Keywords:** colorectal cancer, immune-related gene prognostic index (IRGPI), drug sensitivity, TME, immunotherapy

## Abstract

**Background:**

Colorectal cancer (CRC) is one of the most common solid malignant burdens worldwide. Cancer immunology and immunotherapy have become fundamental areas in CRC research and treatment. Currently, the method of generating Immune-Related Gene Prognostic Indices (IRGPIs) has been found to predict patient prognosis as an immune-related prognostic biomarker in a variety of tumors. However, their role in patients with CRC remains mostly unknown. Therefore, we aimed to establish an IRGPI for prognosis evaluation in CRC.

**Methods:**

RNA-sequencing data and clinical information of CRC patients were retrieved from The Cancer Genome Atlas (TCGA) and The Gene Expression Omnibus (GEO) databases as training and validation sets, respectively. Immune-related gene data was obtained from the *ImmPort* and *InnateDB* databases. The weighted gene co-expression network analysis (WGCNA) was used to identify hub immune-related genes. An IRGPI was then constructed using Cox regression methods. Based on the median risk score of IRGPI, patients could be divided into high-risk and low-risk groups. To further investigate the immunologic differences, Gene set variation analysis (GSVA) studies were conducted. In addition, immune cell infiltration and related functional analysis were used to identify the differential immune cell subsets and related functional pathways.

**Results:**

We identified 49 immune-related genes associated with the prognosis of CRC, 17 of which were selected for an IRGPI. The IRGPI model significantly differentiates the survival rates of CRC patients in the different groups. The IRGPI as an independent prognostic factor significantly correlates with clinico-pathological factors such as age and tumor stage. Furthermore, we developed a nomogram to improve the clinical utility of the IRGPI score. Immuno-correlation analysis in different IRGPI groups revealed distinct immune cell infiltration (CD4^+^ T cells resting memory) and associated pathways (macrophages, Type I IFNs responses, iDCs.), providing new insights into the tumor microenvironment. At last, drug sensitivity analysis revealed that the high-risk IRGPI group was sensitive to 11 and resistant to 15 drugs.

**Conclusion:**

Our study established a promising immune-related risk model for predicting survival in CRC patients. This could help to better understand the correlation between immunity and the prognosis of CRC providing a new perspective for personalized treatment of CRC.

## Introduction

Colorectal cancer (CRC) ranks as the third most prevalent cancer worldwide and is the second leading cause of cancer-related deaths. In 2023, it is estimated that there will be approximately 153,020 new cases of CRC and 52,550 deaths from CRC in the United States (1). Despite the introduction of screening colonoscopy, which has reduced morbidity and mortality, around 25% of CRC patients are initially diagnosed with metastatic disease, and an additional 25% will develop metastases in the course of their illness (2, 3). Currently, surgery remains the primary treatment approach for most CRC cases, with a higher likelihood of achieving permanent remission, particularly in the early stages of the disease. However, for patients with advanced CRC, the 5-year survival rate remains below 10% (1). Despite continuous advancements in the field, including the availability of modern combination treatment options such as surgery, chemotherapy regimens, and immunotherapy, the overall prognosis for CRC patients remains unsatisfactory (4, 5).

Throughout cancer development, various mechanisms have evolved to evade immune surveillance and suppress anti-tumor immune responses. Immune checkpoint pathways play a crucial role in tumor immune evasion. Normally, immune checkpoint molecules regulate immune responses by stimulating or suppressing immune reactions to control infections. However, these immune checkpoint interactions also contribute to cancer pathogenesis, and numerous studies have focused on targeting these interactions to enhance anti-tumor immunity (6, 7). Immune checkpoint blockade (ICB) is an immunotherapy approach that has demonstrated sustained benefits and significantly improved disease outcomes in select patients by reactivating the immune system against cancer cells (8). Immunotherapy has been widely employed in cancer treatment and has shown remarkable efficacy, particularly in lung cancer (9, 10). Moreover, the inhibition of programmed death-1 (PD-1) or programmed cell death-ligand 1 (PD-L1) has already shown improvements in the treatment of CRC. For instance, pembrolizumab, a PD-1 receptor inhibitor, has been approved as a second-line therapy for microsatellite instability-high (MSI-H) CRC (11).

In addition, tumor mutation burden (TMB) has emerged as a significant marker for immunotherapy. Notably, patients harboring microsatellite-stable DNA polymerase epsilon (POLE) mutations have demonstrated durable responses to immunotherapy and long-term disease control, indicating their potential as favorable candidates (12). Moreover, the favorable response observed in patients with high TMB and microsatellite instability (MSI) further validates their utility as predictive biomarkers for immunotherapy (13). TMB measurement methods require high-throughput sequencing technology and large-scale data analysis, which makes them costly and relatively complex. This actually limit their widespread use in clinical practice.

The generation of Immune-Related Gene Prognostic Indices (IRGPIs) has emerged as a promising method for predicting patient prognosis and serving as an immune-related prognostic biomarker in various tumor types (14, 15). However, their role in patients with colorectal cancer (CRC) remains uncertain. In this study, we aimed to investigate the prognostic value of immune-related genes (IRGs) in CRC using a dataset composed of samples from TCGA, GEO, InnateDB, and ImmPort databases. Our primary objective was to identify specific IRGs that exhibit significant prognostic value and construct a CRC-specific Immune-Related Gene Prognostic Index (IRGPI). By developing this prognostic index, our intention was to provide clinicians with a valuable tool to predict patient outcomes and guide personalized treatment approaches.

## Methods

### Sample information collection

RNA sequencing and clinical data for the training sets were downloaded from the TCGA database derived from 568 CRC samples and 44 normal samples. Microarray and clinical data for the validation sets were downloaded from the GEO database derived from 70 CRC samples (GSE39084). In addition, gene mutation data were obtained from the TCGA database. The list of IRGs was obtained from InnateDB (www.innateDBdb.com) and ImmPort (www.immport.org/shared/home) databases.

### Hub immune-related genes

The transcriptome data from TCGA were compiled and normalized and then subjected to differential gene analysis using R’s “*limma*” package. The data were screened using | log_2_ fold change | > 0.585 (fold change > 1.5) and false discovery rate (FDR) < 0.05 as cutoff values to obtain differentially expressed genes (DEGs) (16). By merging the IRGs and the DEGs, differentially expressed immune-related genes (DEIRGs) were identified. Heatmaps of the DEGs and DEIRGs were obtained by visualizing and analyzing the expression levels of the differential genes using the “*pheatmap*” package in R. Gene Ontology (GO) and Kyoto Encyclopedia of Genes and Genomes (KEGG) analysis of DEIRGs were performed using the “*clusterProfiler*” package of R software to study the biological functional changes in CRC samples compared to normal tissue samples (17). To further identify the hub gene modules, we constructed a co-expression network using the weighted gene co-expression network analysis (WGCNA). The following steps were performed: 1. Data preparation: The DEIRGs expression data were processed to remove missing values, outliers, and redundant data. 2.Sample clustering: Hierarchical clustering method was used to cluster the samples and detect outlier samples. 3.Gene co-expression network construction: The “*pickSoftThreshold*” function was used to calculate the power value. Scatter plots of fit indices *vs.* power value and mean connectivity *vs.* power value were plotted to assess the network’s fit and connectivity. The adjacency matrix of the gene co-expression network was built based on the optimal power value (18). The TOM (Topological Overlap Matrix) method was used to calculate the similarity between genes. Gene clustering analysis was performed to construct the gene clustering tree. 4.Module identification and merging: The “*cutreeDynamic*” function was used to perform dynamic tree cutting on the gene clustering tree, assigning genes to different modules. Similar modules were merged into a new module based on their similarity. 5.Module-clinical data association: The correlation between module eigengenes and clinical data was calculated, and a heatmap was generated to visualize the degree of association between modules and different traits.

### Establishment and validation of the prognostic model

Based on the best cutoff value determined by the “*Survminer*” and “*survival*” packages (19), we identified the genes within modules significantly associated with overall survival (OS). IRGPI was constructed by multivariate Cox regression based on IRGs. The IRGPI risk score was obtained by multiplying the expression of a specific gene in the sample by its weight in the Cox model and then summing it. The patients were divided into high- and low-risk IRGPI groups, based on the median gene expression of IRGPI. In the TCGA and GEO cohorts, Kaplan-Meier (K-M) curves were used to show the predictive power of the different groups. Further validation of the prognostic value of IRGPI by univariate and multifactorial Cox regression analysis. Using the “*pheatmap*” package to plot risk score curves. Using the receiver operating characteristic (ROC) curve and the area under the curve (AUC) by the “*survival ROC*” package, the model’s sensitivity and specificity were evaluated to compare the prognostic value among IRGPI, tumor inflammation signature (TIS), and tumor immune dysfunction and exclusion (TIDE). The TIDE score was calculated online (http://tide.dfci.harvard.edu/) to assess the likelihood of tumor immune escape in the gene expression profile of tumor samples. As a biomarker to predict the response of patients with different types of cancer, the TIS score was calculated as the mean of the log_2_-scale normalized expression of 18 signature genes to reflect the extent of immune cell infiltration in the tumor microenvironment.

### IRGPI and clinical characteristics

We explored the correlation between IRGPI and clinic-pathological factors (immune subtype, gender, age, and TNM stage) using the “ComplexHeatmap” packages (20) to investigate the clinical application value. Using the “*rms*” and “*survivor*” packages, a nomogram model was created to estimate risk factors and survival years for CRC patients based on IRGPI risk scores and different clinical parameters (21).

### GSVA and gene mutations

To enable the identification of biological processes and pathways associated with the IRGPI, an enrichment analysis was performed. This analysis was performed using the gene set variation analysis (GSVA) through the “*GSVA*” packages. Finally, we analyzed the mutations of the immune-related genes by the “*maftools*” package to reveal relevant genetic alterations in different IRGPI groups (22).

### Immune cell infiltration and immune function

To further investigate the tumor microenvironment of CRC, the expression data were imported into CIBERSORT (http://cibersort.stanford.edu/) which is an analytical tool developed by Newman et al. (23) that uses gene expression data to estimate the abundance of member cell types. The data was simulated 1000 times. The relationship between IRGPI and immune cell infiltration was then explored by comparing the abundance ratios of the immune cell subsets in each sample. The correlation between the immune cell abundance ratio and OS was analyzed by K-M curves. To further investigate the pathogenic mechanisms of immune differences, we obtained scores of immune-related functions for each sample by ssGSEA analysis to explore the relationship between IRGPI and immune-related pathway activities.

### Drug resistance analysis

Using the “*OncoPredict*” package in R, gene expression profiles of tissues were fitted to the half-maximal inhibitory concentrations (IC50) of cancer cell lines to build a predictive model to determine drug susceptibility in CRC. Gene expression and drug sensitivity data for the training set were obtained from the Genomics of Drug Sensitivity in Cancer (GDSC) database (https://www.cancerrxgene.org/). We analyzed the relationship between IRGPI expression and sensitivity to chemotherapy and targeted drugs. When an anti-tumor drug demonstrates the ability to effectively inhibit and eradicate a tumor at a low dosage, it indicates that the tumor is “sensitive” to the drug. This sensitivity is reflected by a low drug sensitivity score and a favorable anti-cancer response.

### Statistical analysis

R 4.2.1 was used for all statistical analyses and visualizations. For continuous variables, both parametric and non-parametric tests were utilized. Independent Student’s t-tests were initially conducted between the two groups. Normality of the data was assessed using the Kolmogorov-Smirnov test. If the data followed a normal distribution, the results of the t-test were reported. However, if the data did not meet the assumption of normality, the Kruskal-Wallis test, a non-parametric alternative, was used as a global test to evaluate differences among multiple groups before conducting pairwise comparisons. For categorical data, the chi-square test was employed to examine group differences. Prior to conducting pairwise comparisons, the ANOVA test was performed as a global test. Additionally, the Kolmogorov-Smirnov test was applied to assess the assumption of normality for continuous variables within each subgroup. Univariate survival analysis was conducted using the log-rank test and the Kaplan-Meier curve survival analysis. To further explore the impact of potential confounding factors, multivariate survival analysis was performed using Cox regression models. To address the issue of multiple comparisons, the Benjamini-Hochberg procedure was implemented to adjust p-values and control the false discovery rate (FDR). P-values < 0.05 were considered statistically significant.

## Results

### Hub immune-related genes

**Figure 1:**
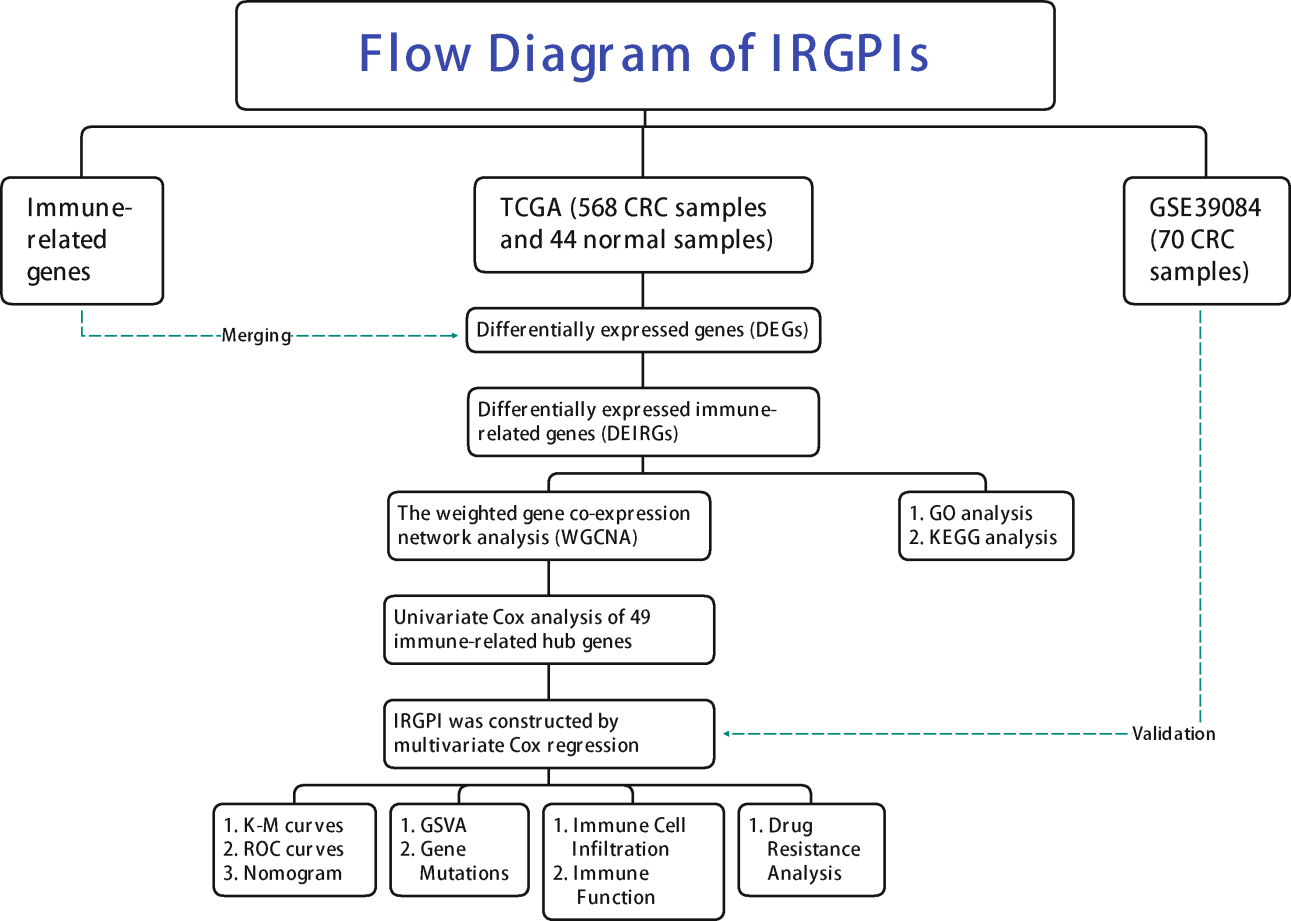
Flow Diagram of IRGPIs.

**Figure 2:**
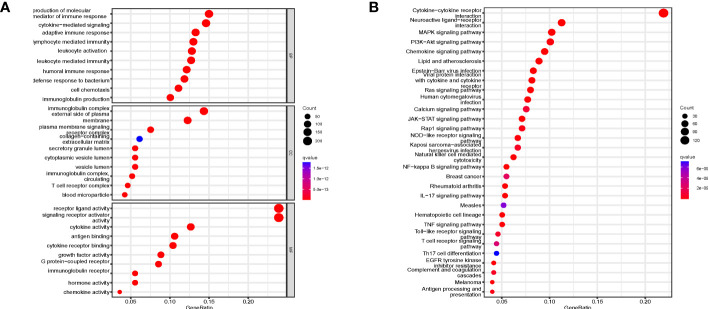
GO and KEGG enrichment analysis. **(A)** GO enrichment analysis of DEIRGs for BP, CC, and MF, respectively. **(B)** KEGG enrichment analysis of DEIRGs. DEGs, differentially expressed genes; DEIRGs, differentially expressed immune-related genes; GO, Gene Ontology; BP, biological process; CC, cellular component; MF, molecular function; KEGG, Kyoto Encyclopedia of Genes and Genomes.

Flow Diagram of IRGPIs is presented in **Figure 1**. A total of 14,767 DEGs were screened by differential analysis of 568 tumors and 44 normal tissue samples from the TCGA database (**Supplementary Figure 1A**). A total of 1,080 DEIRGs were obtained by taking the intersection of the DEGs with the IRGs (**Supplementary Figure 1B**). The top 10 terms of GO are displayed in **Figure 2A**. The most relevant pathways for immune regulation in the biological process (BP), cellular component (CC), and molecular function (MF) domains were the production of molecular mediators of the immune response as well as immunoglobulin complex and receptor ligand activity, respectively. The top 30 terms of KEGG are displayed in **Figure 2B**. The three most relevant pathways for immunity were cytokine−cytokine receptor interaction, neuroactive ligand−receptor interaction, and the MAPK signaling pathway.

**Figure 3:**
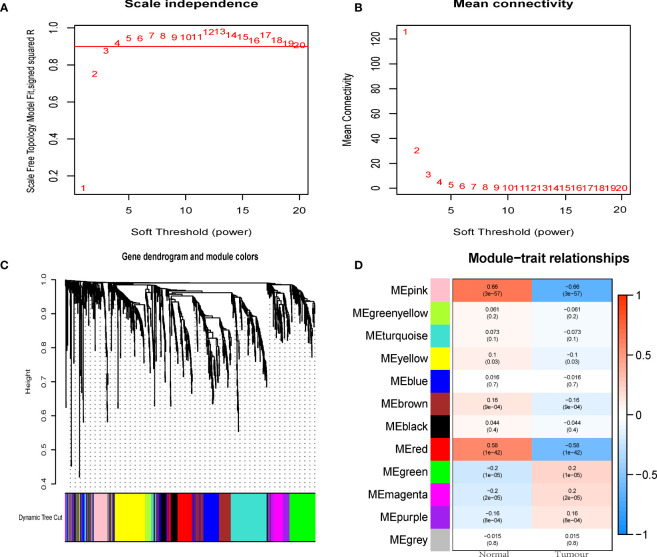
WGCNA to mine differential immune gene modules. **(A)** Scale-independence of various soft-thresholding powers. **(B)** Mean connectivity analysis of various soft-thresholding powers. **(C)** Identification of co-expression modules. **(D)** Heatmap of module-tumour status correlation. WGCNA, weighted gene co-expression network.

We used WGCNA to analyze candidate genes and extract immune-related hub gene modules. The optimal power value was 3 based on the scale independence and mean connectivity (**Figures 3A, B**). From 1080 DEIRGs the dendrogram identified co-expressed gene modules (**Figure 3C**). The final 12 modules were generated by gene clustering and merging similar modules (**Figure 3D**). Based on Pearson correlation coefficients between the sample characteristics and each module, a total of 7 out of 12 modules could distinguish between CRC and normal tissue samples (**Figure 3D**).

### Establishment and validation of the prognostic model

**Figure 4:**
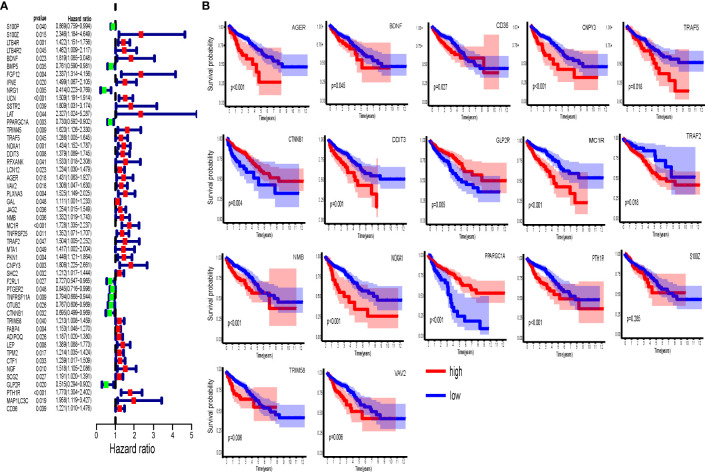
Immune-related hub genes. **(A)** Univariate Cox analysis of 49 immune-related hub genes. **(B)** Kaplan-Meier survival analysis of 17 immune-related hub genes involved in prognostic model construction.

We performed further survival analysis of the genes in the seven modules. Univariate analysis suggests that the expression of 49 hub immune-related genes is highly correlated with OS (**Figure 4A**). Based on multivariate Cox regression analysis, 17 genes have significant impact on the OS of CRC patients and were therefore selected for the construction of prognostic models (**Figure 4B**). The calculation formula is as follows: IRGPI = S100Z × 1.016 + BDNF × 0.625 − PPARGC1A × 0.405 + TRAF5 × 0.370 + NOXA1 × 0.494 + DDIT3 × 0.249 − AGER × 0.507 + VAV2 × 0.365 + NMB × 0.300 + MC1R × 0.430 − TRAF2 × 0.830 + CNPY3 × 0.535 − CTNNB1 × 0.555 + TRIM58 × 0.232 − GLP2R × 0.906 + PTH1R × 0.404 + CD36 × 0.289.

**Figure 5:**
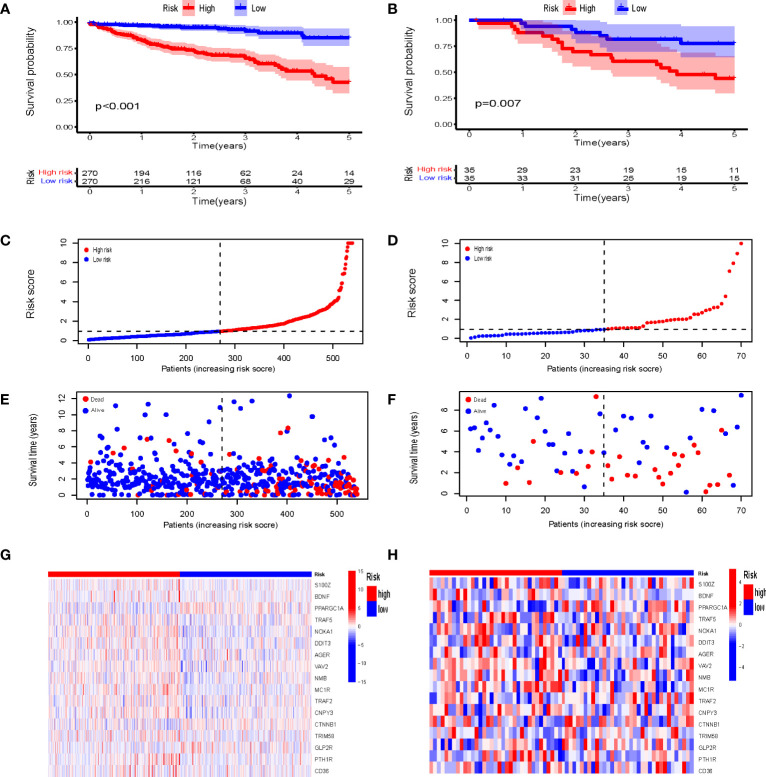
Establishment and Validation of the Prognostic Model. Kaplan-Meier survival analysis of IRGPI groups in TCGA cohort **(A)** and GEO cohort **(B)**. Risk score distribution of gene signatures based on TCGA cohort **(C)** and GEO cohort **(D)**. Survival status of gene signatures based on TCGA cohort **(E)** and GEO cohort **(F)**. Heatmaps of the differential expression of metabolic genes in high-risk and low-risk groups based on TCGA cohort **(G)** and GEO cohort **(H)**. TCGA, The Cancer Genome Atlas; and GEO, Gene Expression Omnibus.

**Figure 6:**
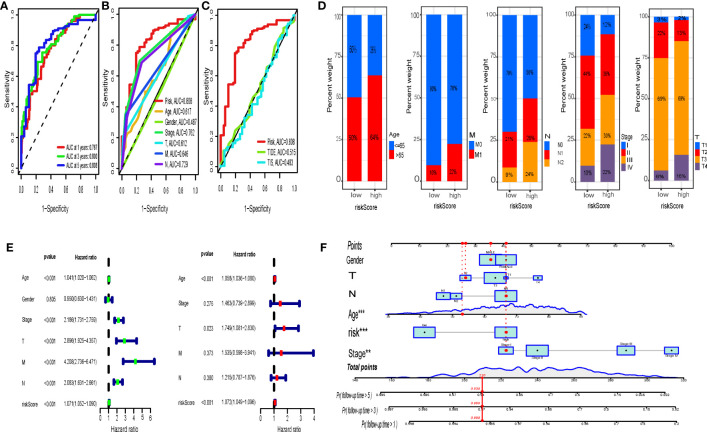
IRGPI and ROC curve analysis and clinical characteristics. **(A)** Time-dependent ROC curve analysis of IRGPI at 1, 3, and 5 years. The comparison of this model with clinicopathological factors **(B)** and TIDE, TIS **(C)** by the time-dependent ROC curve. **(D)** Analysis of the constituent ratio of clinical characteristics in the different IRGPIs. **(E)** Univariate Cox analysis of clinicopathological factors and IRGPI scores, and multivariate Cox analysis of factors significant in univariate Cox analysis (P < 0.05). **(F)** Nomogram containing the risk score to predict the overall survival in CRC patients. IRGPI, Immune-Related Gene Prognostic Index; ROC, receiver operating characteristic.

Both the K-M curve, risk score distribution, and survival status (**Figures 5A, C, E**) in the TCGA training sets display that the low-risk IRGPI group has a better OS (p < 0.001). The analysis from the GSE39084 (n = 70) validation sets shows that CRC in the low-risk IRGPI group has a significantly better OS compared to the high-risk IRGPI group (**Figures 5B, D, F**), which was consistent with the results of the TCGA training sets. The heatmaps illustrate the expression of immune genes in different groups (**Figures 5G, H**). Furthermore, the AUCs for 1-, 3-, and 5-year survival were 0.767, 0.800, and 0.808, by ROC curve analysis, respectively (**Figure 6A**). We compared the risk score of IRGPI with other clinical indicators. The AUCs for the IRGPI risk, TNM stage, N, M, age, T, and gender were 0.808, 0.762, 0.729, 0.646, 0.617, 0.612, and 0.487, respectively (**Figure 6B**). In addition, we compared the predictive performance of the IRGPI with widely used immune-related biomarkers, and the IRGPI achieved superior performance with an AUC of 0.808 in predicting 5-year OS, which suggested that IRGPI was a more reliable signature compared to the AUC of TIDE (0.515) and TIS (0.483) (**Figure 6C**).

### IRGPI and clinical characteristics

To further test the value of IRGPI for clinical application, we investigated the association between IRGPI and clinicopathological variables (**Supplementary Figure 2**). The IRGPI was able to distinguish well between different ages (≥60, <60), TNM stages, T stages, N stages, and M stages with statistical significance (**Figure 6D**). Using univariate Cox regression analysis, IRGPI and other clinicopathological factors (age, stage, T, N, M) were found to be strongly associated with prognosis in CRC (**Figure 6E**). In addition, IRGPI was an independent prognostic factor in the multifactorial Cox regression analysis (**Figure 6E**). Furthermore, based on the IRGPI scores and clinical characteristics, we developed a new nomogram to predict the survival of CRC patients. This nomogram can effectively predict the probability of CRC patients’ overall survival probability of 1 year, 3 years, and 5 years (**Figure 6F**).

### GSVA and gene mutations

**Figure 7:**
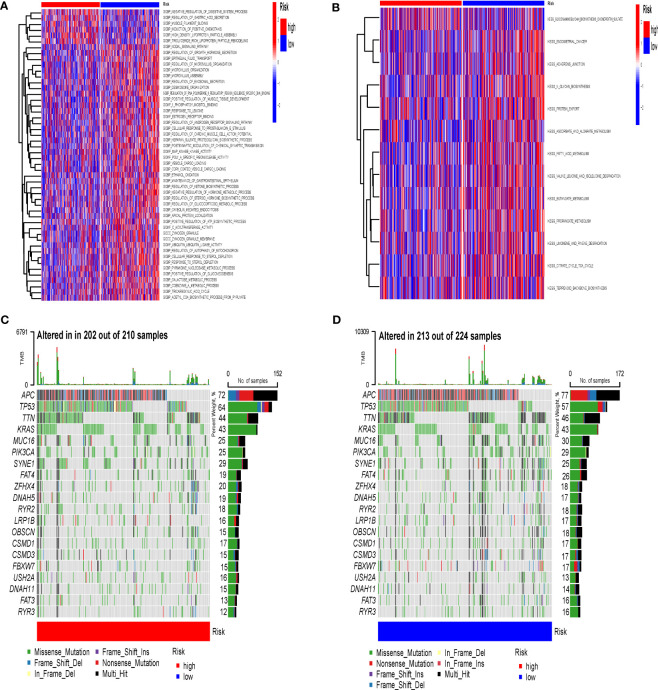
GSVA and Gene Mutations. **(A)** Enrichment analysis of GSVA in IRGPI groups (C2 Gene Set). **(B)** Enrichment analysis of GSVA in IRGPI groups (C5 Gene Set). **(C)** Significantly mutated genes in high-risk IRGPI groups. **(D)** Significantly mutated genes in high-risk IRGPI groups. GSVA, Gene Set Variation Analysis; IRGPI, Immune-Related Gene Prognostic Index.

In this study, we used GSVA to explore the changes in immune function and pathways between different IRGPI groups. The GSVA analysis was performed using two gene sets: C2 Gene Set, which contains KEGG information, and C5 Gene Set, which contains GO (Gene Ontology) information. For the C2 Gene Set, the analysis revealed that the differential pathways enriched in the high and low risk groups were primarily linked to the negative regulation of digestive system process, regulation of gastric acid secretion, and maintenance of gastrointestinal epithelium (**Figure 7A**). It implies that the high and low risk groups may have distinct molecular mechanisms and regulatory processes governing their digestive system functions. For the C5 Gene Set, the results showed that the differential pathways enriched in the high and low risk groups were mainly associated with propanoate metabolism, fatty acid metabolism, citrate cycle tca cycle, and butanoate metabolism (**Figure 7B**). It implies that individuals in the high and low risk groups may exhibit different metabolic profiles and functional activities, particularly in terms of propionate metabolism, fatty acid metabolism, and related pathways.

To explore immune differences in IRGPI groups at the molecular level, we analyzed gene mutations (**Figures 7C, D**). The results demonstrate that missense mutation was the most common mutation type in CRC. The three most common mutated genes was APC. In addition, the overall mutation rate was slightly higher in the high-risk group compared to the low-risk group.

### Immune cell infiltration and immune function

We analyzed the differences in 22 immune cell infiltration patterns and 29 immune signaling pathways between the two groups. The immune cell proportions of the different IRGPI groups are presented in **Supplementary Figure 3A**. The abundance of CD4^+^ T cells resting memory (TRM) was higher in the low-risk IRGPI group, while other immune cells did not differ significantly in the two groups.

The K-M curves show that the proportions of five immune cell subsets (resting dendritic cells, native B cells, M0 macrophages, resting master cells, and resting NK cells) are associated with the OS (**Supplementary Figures 3B–F**). Among them, higher abundance ratios of resting M0 macrophages, resting NK cells, and naive B cells were associated with shorter OS, whereas higher abundance ratios of resting master cells and resting dendritic cells were associated with longer OS.

Immune function analysis demonstrated that the macrophages and type I interferon responses are associated with the high-risk IRGPI group, whereas the dendritic cells are associated with the low-risk IRGPI group (**Supplementary Figure 3G**).

### Drug resistance analysis

The gene expression data from GDSC and drug sensitivity data for 198 drugs were analyzed and modeled using “OncoPredict” to predict drug sensitivity in CRC samples. We compared the differences in drug sensitivity between different risk groups and observed significant variations in drug response between high- and low-risk IRGPI group for 26 chemotherapeutic drugs. Among these drugs, the high-risk IRGPI group showed sensitivity to 11 drugs, including dasatinib, rapamycin, and taselisib, while the low-risk IRGPI group exhibited sensitivity to 15 drugs, including bortezomib, sorafenib, and ulixertinibin (**Supplementary Figure 4**).

## Discussion

With the development of cancer immunotherapy and targeted drugs, treatment of advanced and especially metastatic CRC has shifted from traditional surgery and radiotherapy to precise, individualized treatment (24, 25). Although some studies have constructed models based on copper metabolism-related (26), cuproptosis-related (27) genes to guide personalized treatment of CRC, which are still in the exploratory stage. Accurate CRC classification can help improve patient survival by guiding personalized treatment in the clinical setting. The immune system plays a key role in tumorigenesis, influencing the immune response, immune evasion, and immunotherapy. Regulation of the immune system is essential for maintenance of tissue homeostasis and is a key factor in cancer development and progression (28, 29). Based on the role of immune-related genes in tumors, the prognostic role of different IRGPI classifications in other tumors (breast cancer (14), hepatocellular carcinoma (21), lung adenocarcinoma (15), melanoma (30), etc.) has been demonstrated. Lin et al. (31) found a strong correlation between the genes of the IRGPI and previously identified prognostic and clinically relevant indicators (OS, TNM stage, gender, age). In addition, Liang et al. (32) illustrated that the interaction of immune-related genes based on IRGPI calculations may influence CRC formation and development. In contrast, more genes were included in our study with better predictive accuracy (the AUCs for 1-, 3-, and 5-year survival were 0.767, 0.800, 0.808 vs. 0.723, 0.724, 0.744). The IRGPI classification can be used to better understand the prognostic characteristics of tumors, providing clinicians with a useful tool to help guide decisions.

In this study, we developed an IRGPI, which can be used to assess prognosis and immune response in CRC patients and may become a new biomarker for cancer research. We identified 49 immune-related key genes that affect OS by WGCNA. Based on 17 of these genes we constructed a predictive model for CRC. In the TCGA and GEO cohorts, IRGPI scores were inversely correlated with survival. Currently, the TNM stage reflects the extent of cancer spread and is widely used as a clinical guide to assessing the degree of tumor progression and aggressiveness after surgery, as well as the prognosis of patients (33, 34). However, Patients with the same tumor stage had significantly different clinical outcomes (35). This demonstrates the limitations of the TNM staging system. Using ROC curve analysis, IRGPI has better reliability and validity in predicting survival compared to the TNM stage. The IRGPI also had better accuracy compared to other indicators (TIDE, TIS). Lin et al. (31) constructed an IRGPI prediction model based on the TCGA database with an AUC of 0.858, however, no other database was used to validate the developed prediction model. In addition, IRGPI, as a prognostic indicator, was closely correlated with pathological factors (age, stage, T, N, M), and participated in the construction of a nomogram, which allows for a feasible clinical application.

We performed an enrichment analysis to understand the biological processes and pathways associated with IRGPI. Differential immune genes were associated with the following pathways: tumor-related MAP kinase activity, zymogen granule, fatty acid metabolism, and butanoate metabolism. In previous studies, the nodal signaling pathway (36) has been demonstrated to be an important pathway involved in tumorigenesis and metastasis. Meanwhile, MAP kinase activity (37), zymogen granule (38), fatty acid metabolism (39), and butanoate metabolism (40) also play a vital role in tumor progression.

Furthermore, we analyzed gene mutations to explore immune differences in IRGPI at the molecular level. Although this difference did not reach statistical significance, it is worth noting that factors such as sample size and effect size might have contributed to this non-significant result. Moreover, the mutation rate can be influenced by various factors including tumor type, genetic heterogeneity, and environmental factors. Therefore, it is important to consider these additional factors when interpreting the observed mutation rate (41). Among the frequently mutated genes, APC was the most commonly observed (42). APC mutations are present in 80% of CRC cases and play a role in the initiation of colorectal adenoma development (43, 44).

Considering the importance of TME in tumorigenesis, development, and treatment, we assessed the relative proportions of immune cells and the immune function in different IRGPI groups. Here, we found that IRGPI was associated with the infiltration of CD4^+^ TRM. The relative ratios of resting dendritic cells, resting mast cells, M0 macrophages, resting NK cells, and naive B cells were significantly correlated with the OS of CRC. These immune cells make a significant contribution to the prognosis of CRC. For example, infiltration of CD4^+^ TRM increased the anti-tumor activity of tissues by promoting the recruitment of immune cells and was associated with a good prognosis of tumors (45). Macrophages promote immune evasion and tumor invasion by secreting anti-inflammatory cytokines and increasing the expression of MHC-like molecules (46). In addition, CD8+ T cells, dendritic cells, natural killer T cells, and natural killer cells constituted a line of defense against tumors by secreting pro-inflammatory cytokines, producing reactive oxygen species, and mediating cytotoxic effects (47).

Currently, CRC is treated mainly by surgery as well as conventional chemotherapy, and only selected patients benefit from immunotherapy. However, chemotherapeutic drugs enhance the effectiveness of immunotherapy by altering the tumor microenvironment. Therefore, it is necessary to explore the sensitivity of different IRGPI groups to chemotherapeutic drugs. Studies of the sensitivity of patients in different IRGPI groups to chemotherapeutic agents showed that the high-risk IRGPI group was sensitive to 11 drugs and resistant to 15 drugs. Among them, the combination of dasatinib and curcumin inhibited the growth and invasion of chemo-resistant colon tumor cells (48). Wang et al. certificated that the combination of bortezomib and leucovorin promoted CRC cell apoptosis and inhibited tumor growth and can be used in the treatment of CRC (49). Cetuximab, a promising antineoplastic agent, has been used to treat patients with BRAFV600E-mutated colorectal cancer (50). In addition, studies evaluating the efficacy and safety of rapamycin, sorafenib, taselisib, and ulixertinibin patients with CRC are in the clinical phase. Sparse data are available at present, but identifying individualized therapeutic approaches based on IRGPI subgroups may be beneficial for the improvement of the efficacy of CRC therapies in the future.

Despite a comprehensive analysis of immune-related genes, our study has some limitations. First, the data were obtained from different public databases with different inclusion and exclusion criteria, which may be subject to selective bias and a limited number of studies. Therefore, the conclusions of the study need to be validated with further prospective, large-scale, multicenter data. Second, the transcriptome analysis reflects only some aspects of the immune status of CRC, and we plan to expand our studies in metabolomics, proteomics, and genomics to provide a more comprehensive understanding of CRC immunity. Third, the IRGPI model we developed can be used to predict patient survival; however, the mechanisms by which gene interactions in the model affect tumor progression have not been investigated. In addition, the number of genes constituting the model is high, which is not conducive to clinical application and needs to be further optimized.

In summary, our study established a promising immune-related risk model for predicting survival in CRC patients. This is the first WGCNA-based prognostic model of immune-related genes that will help researchers better understand the correlation between immunity and the prognosis of CRC and provide a new perspective for personalized treatment of CRC.

## Data availability statement

The datasets presented in this study can be found in online repositories. The names of the repository/repositories and accession number(s) can be found within the article.

## Author contributions

CL, UW and FK designed research, analyzed data, and wrote the paper. All authors contributed to the article and approved the submitted version.

## Supplementary material

Supplementary Figure 1 Screening for DEGs and enrichment analysis. **(A)** Heatmap of DEGs. **(B)** Heatmap of DEIRGs.

Supplementary Figure 2 IRGPI and Clinical Characteristics.

Supplementary Figure 3 Immune Cell Infiltration and Immune Function. **(A)** The correlation of IRPGI scores with 22 immune cells. **(B–F)** Kaplan-Meier survival analysis of the correlation of immune cell abundance ratios in the IRGPI groups. IRGPI, immune-related gene prognostic index. **(G)** The correlation of IRPGI scores with 29 immune signaling pathways.

Supplementary Figure 4 Drug sensitivity analysis.

## References

[1] Siegel RL Wagle NS Cercek A Smith RA Jemal A. Colorectal cancer statistics, 2023. CA Cancer J Clin (2023) 73(3):233–54. doi: 10.3322/caac.21772 36856579

[2] Pinsky PF Doroudi M. Colorectal cancer screening. JAMA (2016) 316(16):1715. doi: 10.1001/jama.2016.13849 27784086

[3] Edwards BK Ward E Kohler BA Eheman C Zauber AG Anderson RN. Annual report to the nation on the status of cancer, 1975-2006, featuring colorectal cancer trends and impact of interventions (risk factors, screening, and treatment) to reduce future rates. Cancer (2010) 116(3):544–73. doi: 10.1002/cncr.24760 PMC3619726 19998273

[4] Morris VK Kennedy EB Baxter NN Benson AB 3rd Cercek A Cho M. Treatment of metastatic colorectal cancer: ASCO guideline. J Clin Oncol (2023) 41(3):678–700. doi: 10.1200/JCO.22.01690 36252154 PMC10506310

[5] Saltz LB. Top advances of the year in colorectal cancer. Cancer (2022) 128(12):2236–9. doi: 10.1002/cncr.34185 35323989

[6] Pardoll DM. The blockade of immune checkpoints in cancer immunotherapy. Nat Rev Cancer (2012) 12(4):252–64. doi: 10.1038/nrc3239 PMC4856023 22437870

[7] Wykes MN Lewin SR. Immune checkpoint blockade in infectious diseases. Nat Rev Immunol (2018) 18(2):91–104. doi: 10.1038/nri.2017.112 28990586 PMC5991909

[8] Morad G Helmink BA Sharma P Wargo JA. Hallmarks of response, resistance, and toxicity to immune checkpoint blockade. Cell (2021) 184(21):5309–37. doi: 10.1016/j.cell.2021.09.020 PMC8767569 34624224

[9] Brahmer J Reckamp KL Baas P Crinò L Eberhardt WE Poddubskaya E. Nivolumab versus docetaxel in advanced squamous-cell non-Small-Cell lung cancer. N Engl J Med (2015) 373(2):123–35. doi: 10.1056/NEJMoa1504627 PMC4681400 26028407

[10] Robert C Reckamp KL Baas P Crinò L Eberhardt WE Poddubskaya E. Ipilimumab plus dacarbazine for previously untreated metastatic melanoma. N Engl J Med (2011) 364(26):2517–26. doi: 10.1056/NEJMoa1104621 21639810

[11] Coupez D Hulo P Touchefeu Y Bossard C Bennouna J. Pembrolizumab for the treatment of colorectal cancer. Expert Opin Biol Ther (2020) 20(3):219–26. doi: 10.1080/14712598.2020.1718095 31952453

[12] Gong J Wang C Lee PP Chu P Fakih M. Response to PD-1 blockade in microsatellite stable metastatic colorectal cancer harboring a POLE mutation. J Natl Compr Canc Netw (2017) 15(2):142–7. doi: 10.6004/jnccn.2017.0016 28188185

[13] Palmeri M Mehnert J Silk AW Jabbour SK Ganesan S Popli P. Real-world application of tumor mutational burden-high (TMB-high) and microsatellite instability (MSI) confirms their utility as immunotherapy biomarkers. ESMO Open (2022) 7(1):100336. doi: 10.1016/j.esmoop.2021.100336 34953399 PMC8717431

[14] Yao Y Kong X Liu R Xu F Liu G Sun C. Development of a novel immune-related gene prognostic index for breast cancer. Front Immunol (2022) 13:845093. doi: 10.3389/fimmu.2022.845093 PMC9086776 35558081

[15] Wang C Lu T Xu R Chang X Luo S Peng B. A bioinformatics-based immune-related prognostic index for lung adenocarcinoma that predicts patient response to immunotherapy and common treatments. J Thorac Dis (2022) 14(6):2131–46. doi: 10.21037/jtd-22-494 PMC9264088 35813746

[16] Ritchie ME Phipson B Wu D Hu Y Law CW Shi W. Limma powers differential expression analyses for RNA-sequencing and microarray studies. Nucleic Acids Res (2015) 43(7):e47. doi: 10.1093/nar/gkv007 25605792 PMC4402510

[17] Yu G Wang LG Han Y He QY. clusterProfiler: an r package for comparing biological themes among gene clusters. OMICS (2012) 16(5):284–7. doi: 10.1089/omi.2011.0118 PMC3339379 22455463

[18] Niemira M Collin F Szalkowska A Bielska A Chwialkowska K Reszec J. Molecular signature of subtypes of non-small-cell lung cancer by Large-scale transcriptional profiling: identification of key modules and genes by weighted gene Co-expression network analysis (WGCNA). Cancers (Basel) (2019) 12(1):37. doi: 10.3390/cancers12010037 31877723 PMC7017323

[19] Lorent M Giral M Foucher Y. Net time-dependent ROC curves: a solution for evaluating the accuracy of a marker to predict disease-related mortality. Stat Med (2014) 33(14):2379–89. doi: 10.1002/sim.6079 24399671

[20] Gu Z Eils R Schlesner M. Complex heatmaps reveal patterns and correlations in multidimensional genomic data. Bioinformatics (2016) 32(18):2847–9. doi: 10.1093/bioinformatics/btw313 27207943

[21] Dai Y Qiang W Lin K Gui Y Lan X Wang D. An immune-related gene signature for predicting survival and immunotherapy efficacy in hepatocellular carcinoma. Cancer Immunol Immunother (2021) 70(4):967–79. doi: 10.1007/s00262-020-02743-0 PMC10992402 33089373

[22] Mayakonda A Lin DC Assenov Y Plass C Koeffler HP. Maftools: efficient and comprehensive analysis of somatic variants in cancer. Genome Res (2018) 28(11):1747–56. doi: 10.1101/gr.239244.118 PMC6211645 30341162

[23] Newman AM Liu CL Green MR Gentles AJ Feng W Xu Y. Robust enumeration of cell subsets from tissue expression profiles. Nat Methods (2015) 12(5):453–7. doi: 10.1038/nmeth.3337 PMC4739640 25822800

[24] Zhao Y Wei K Chi H Xia Z Li X. IL-7: a promising adjuvant ensuring effective T cell responses and memory in combination with cancer vaccines? Front Immunol (2022) 13:1022808. doi: 10.3389/fimmu.2022.1022808 PMC9650235 36389666

[25] Jin W Yang Q Chi H Wei K Zhang P Zhao G. Ensemble deep learning enhanced with self-attention for predicting immunotherapeutic responses to cancers. Front Immunol (2022) 13:1025330. doi: 10.3389/fimmu.2022.1025330 PMC9751999 36532083

[26] Zhao S Zhang X Gao F Chi H Zhang J Xia Z. Identification of copper metabolism-related subtypes and establishment of the prognostic model in ovarian cancer. Front Endocrinol (Lausanne) (2023) 14:1145797. doi: 10.3389/fendo.2023.1145797 PMC10025496 36950684

[27] Li Y Wang RY Deng YJ Wu SH Sun X Mu H. Molecular characteristics, clinical significance, and cancer immune interactions of cuproptosis and ferroptosis-associated genes in colorectal cancer. Front Oncol (2022) 12:975859. doi: 10.3389/fonc.2022.975859 PMC9483209 36132144

[28] Zhao S Chi H Yang Q Chen S Wu C Lai G. Identification and validation of neurotrophic factor-related gene signatures in glioblastoma and parkinson's disease. Front Immunol (2023) 14:1090040. doi: 10.3389/fimmu.2023.1090040 PMC9941742 36825022

[29] Gong X Chi H Strohmer DF Teichmann AT Xia Z Wang Q. Exosomes: a potential tool for immunotherapy of ovarian cancer. Front Immunol (2022) 13:1089410. doi: 10.3389/fimmu.2022.1089410 PMC9889675 36741380

[30] Yan J Wu X Yu J Kong Y Cang S. An immune-related gene pair index predicts clinical response and survival outcome of immune checkpoint inhibitors in melanoma. Front Immunol (2022) 13:839901. doi: 10.3389/fimmu.2022.839901 PMC8907429 35280982

[31] Lin K Huang J Luo H Luo C Zhu X Bu F. Development of a prognostic index and screening of potential biomarkers based on immunogenomic landscape analysis of colorectal cancer. Aging (Albany NY) (2020) 12(7):5832–57. doi: 10.18632/aging.102979 PMC7185108 32235004

[32] Liang Z Sun R Tu P Liang Y Liang L Liu F. Immune-related gene-based prognostic index for predicting survival and immunotherapy outcomes in colorectal carcinoma. Front Immunol (2022) 13:944286. doi: 10.3389/fimmu.2022.944286 PMC9795839 36591255

[33] Locker GY Hamilton S Harris J Jessup JM Kemeny N Macdonald JS. ASCO 2006 update of recommendations for the use of tumor markers in gastrointestinal cancer. J Clin Oncol (2006) 24(33):5313–27. doi: 10.1200/JCO.2006.08.2644 17060676

[34] Weitz J Koch M Debus J Höhler T Galle PR Büchler MW. Colorectal cancer. Lancet (2005) 365(9454):153–65. doi: 10.1016/S0140-6736(05)17706-X 15639298

[35] Hattori A Takamochi K Oh S Suzuki K. New revisions and current issues in the eighth edition of the TNM classification for non-small cell lung cancer. Jpn J Clin Oncol (2019) 49(1):3–11. doi: 10.1093/jjco/hyy142 30277521

[36] Seftor EA Seftor REB Weldon D Kirsammer GT Margaryan NV Gilgur A. Melanoma tumor cell heterogeneity: a molecular approach to study subpopulations expressing the embryonic morphogen nodal. Semin Oncol (2014) 41(2):259–66. doi: 10.1053/j.seminoncol.2014.02.001 PMC4026856 24787297

[37] Salh B Marotta A Matthewson C Ahluwalia M Flint J Owen D. Investigation of the mek-MAP kinase-rsk pathway in human breast cancer. Anticancer Res (1999) 19(1B):731–40. 10216485

[38] Meng H Yao W Yin Y Li Y Ding Y Wang L. ZG16 promotes T-cell mediated immunity through direct binding to PD-L1 in colon cancer. biomark Res (2022) 10(1):47. doi: 10.1186/s40364-022-00396-y 35831911 PMC9281127

[39] Corbet C Pinto A Martherus R Santiago de Jesus JP Polet F Feron O. Acidosis drives the reprogramming of fatty acid metabolism in cancer cells through changes in mitochondrial and histone acetylation. Cell Metab (2016) 24(2):311–23. doi: 10.1016/j.cmet.2016.07.003 27508876

[40] Ma X Mo M Tan HJJ Tan C Zeng X Zhang G. LINC02499, a novel liver-specific long non-coding RNA with potential diagnostic and prognostic value, inhibits hepatocellular carcinoma cell proliferation, migration, and invasion. Hepatol Res (2020) 50(6):726–40. doi: 10.1111/hepr.13491 32039538

[41] Sillo TO Beggs AD Middleton G Akingboye A. The gut microbiome, microsatellite status and the response to immunotherapy in colorectal cancer. Int J Mol Sci (2023) 24(6):5767. doi: 10.3390/ijms24065767 36982838 PMC10054450

[42] Shyr C Tarailo-Graovac M Gottlieb M Lee JJ van Karnebeek C Wasserman WW. FLAGS, frequently mutated genes in public exomes. BMC Med Genomics (2014) 7:64. doi: 10.1186/s12920-014-0064-y 25466818 PMC4267152

[43] Kwong LN Dove WF. APC and its modifiers in colon cancer. Adv Exp Med Biol (2009) 656:85–106. doi: 10.1007/978-1-4419-1145-2_8 19928355 PMC3754875

[44] Zhang L Shay JW. Multiple roles of APC and its therapeutic implications in colorectal cancer. J Natl Cancer Inst (2017) 109(8):djw332. doi: 10.1093/jnci/djw332 28423402 PMC5963831

[45] Song J Wu L. Friend or foe: prognostic and immunotherapy roles of BTLA in colorectal cancer. Front Mol Biosci (2020) 7:148. doi: 10.3389/fmolb.2020.00148 32793631 PMC7385242

[46] Liu Z Gao Z Li B Li J Ou Y Yu X. Lipid-associated macrophages in the tumor-adipose microenvironment facilitate breast cancer progression. Oncoimmunology (2022) 11(1):2085432. doi: 10.1080/2162402X.2022.2085432 35712121 PMC9196645

[47] Guo X Tan S Wang T Sun R Li S Tian P. NAD(+) salvage governs mitochondrial metabolism, invigorating natural killer cell antitumor immunity. Hepatology (2022). doi: 10.1002/hep.32658 35815363

[48] Nautiyal J Kanwar SS Yu Y Majumdar AP. Combination of dasatinib and curcumin eliminates chemo-resistant colon cancer cells. J Mol Signal (2011) 6:7. doi: 10.1186/1750-2187-6-7 21774804 PMC3162943

[49] Wang S Wang L Zhou Z Deng Q Li L Zhang M. Leucovorin enhances the anti-cancer effect of bortezomib in colorectal cancer cells. Sci Rep (2017) 7(1):682. doi: 10.1038/s41598-017-00839-9 28386133 PMC5429730

[50] Huijberts SCFA Boelens MC Bernards R Opdam FL. Mutational profiles associated with resistance in patients with BRAFV600E mutant colorectal cancer treated with cetuximab and encorafenib +/- binimetinib or alpelisib. Br J Cancer (2021) 124(1):176–82. doi: 10.1038/s41416-020-01147-2 PMC7782586 33204026

