# Supplementary figures and images for: An immune-related gene prognostic index for predicting prognosis in patients with colorectal cancer
Source: Front Immunol. 2023 Jul 6;14:1156488. doi: 10.3389/fimmu.2023.1156488 (PMC10358773; doi:10.3389/fimmu.2023.1156488)

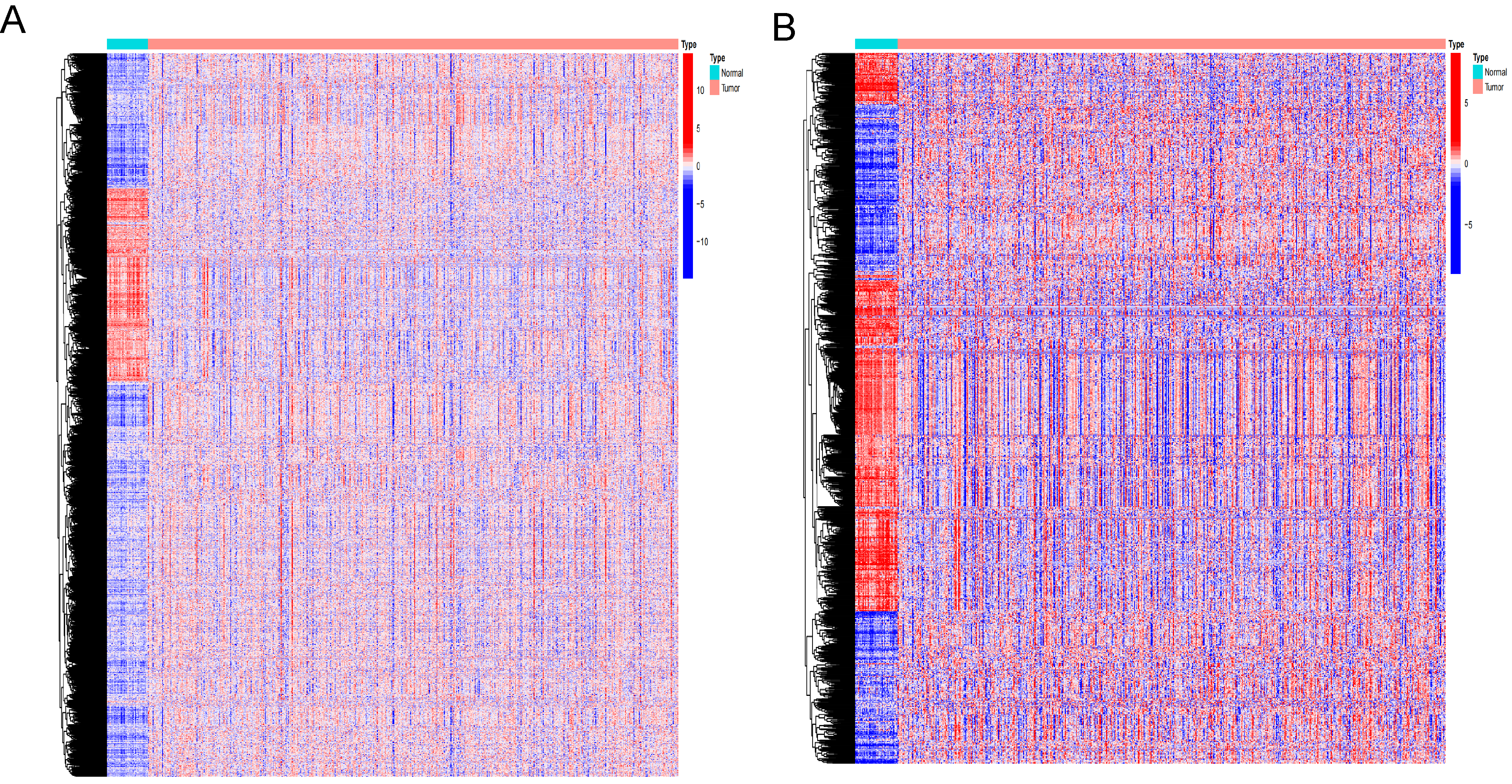

Supplement: Supplementary Figure 1 — Screening for DEGs and enrichment analysis. (A) Heatmap of DEGs. (B) Heatmap of DEIRGs. [file Image_1.tif]

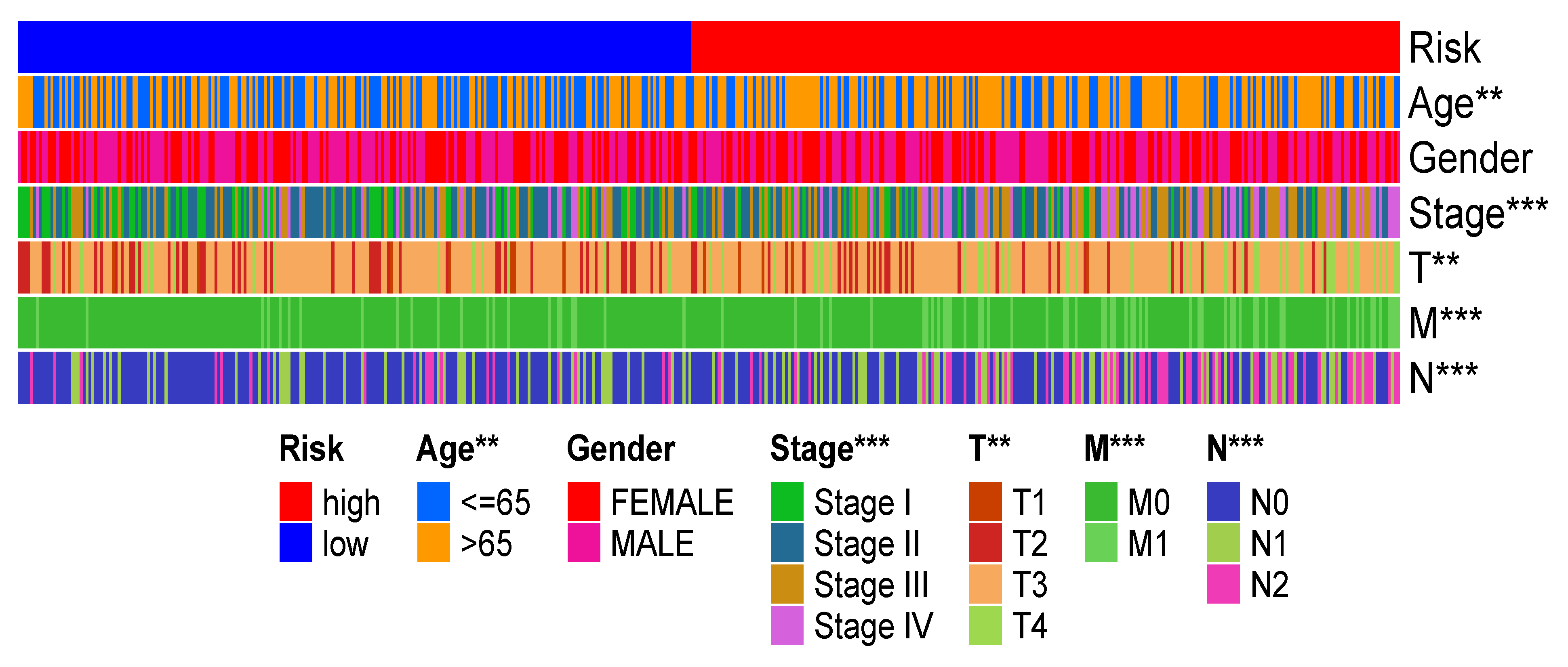

Supplement: Supplementary Figure 2 — IRGPI and Clinical Characteristics. [file Image_2.tif]

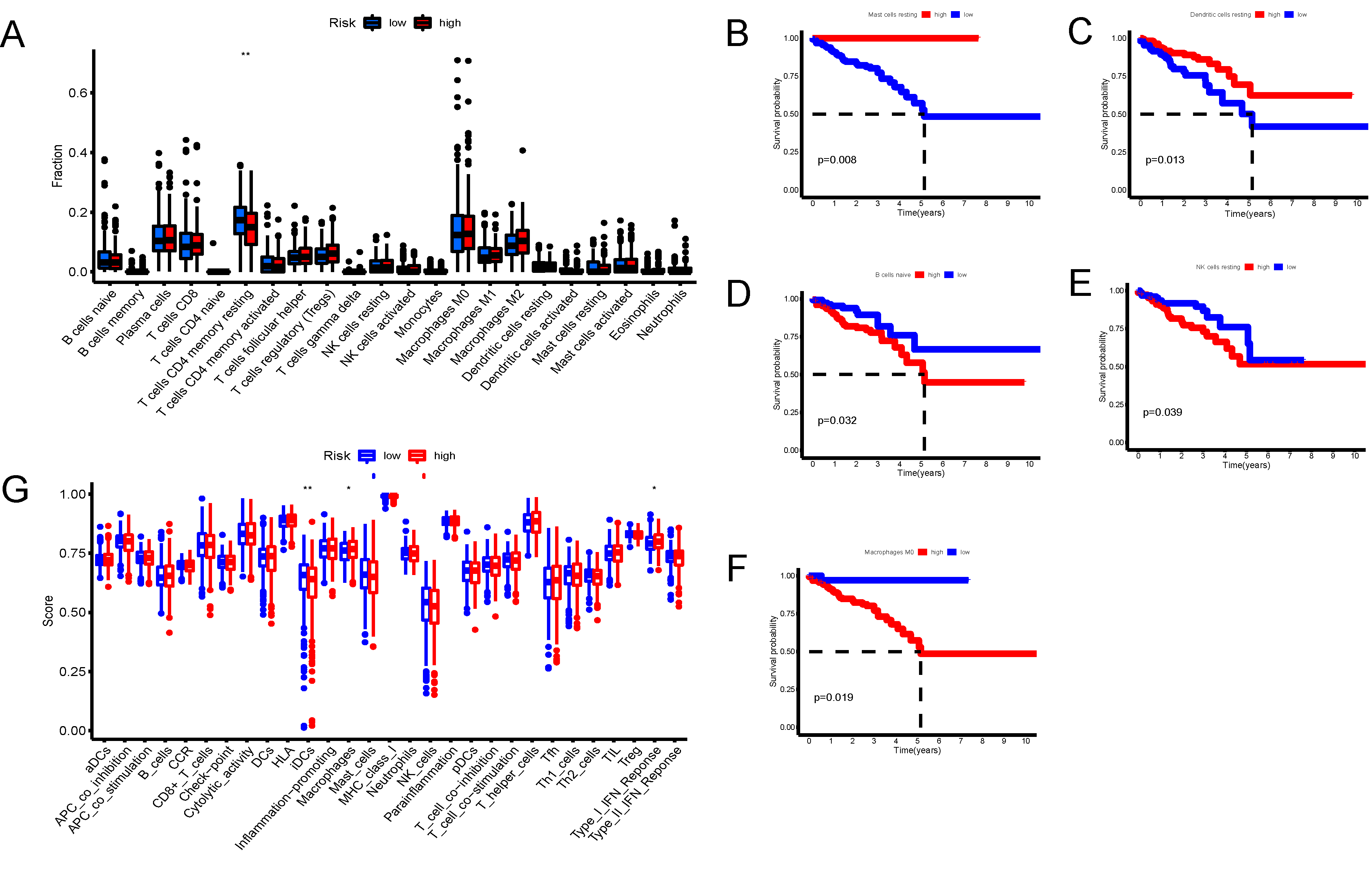

Supplement: Supplementary Figure 3 — Immune Cell Infiltration and Immune Function. (A) The correlation of IRPGI scores with 22 immune cells. (B–F) Kaplan-Meier survival analysis of the correlation of immune cell abundance ratios in the IRGPI groups. IRGPI, immune-related gene prognostic index. (G) The correlation of IRPGI scores with 29 immune signaling pathways. [file Image_3.tif]

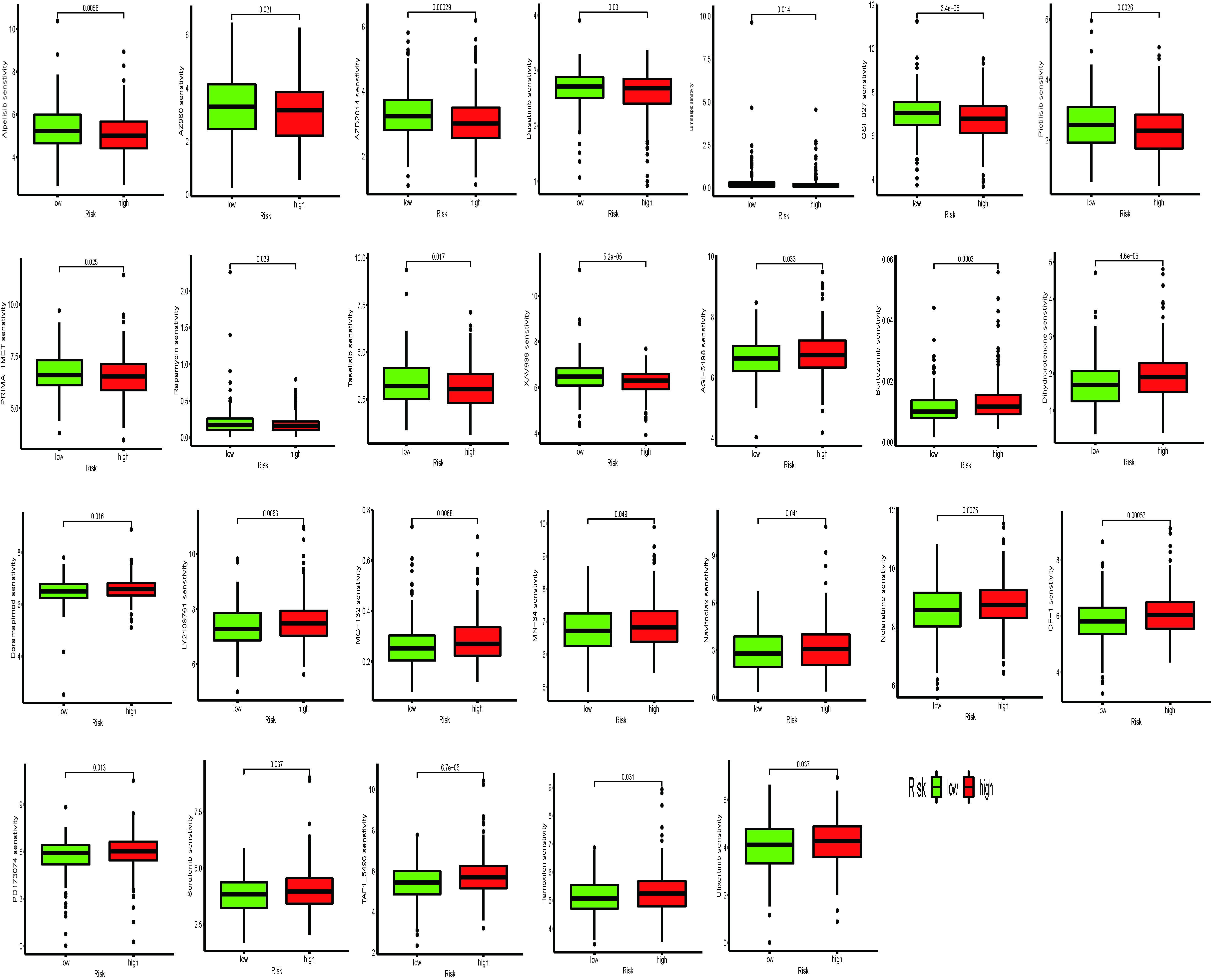

Supplement: Supplementary Figure 4 — Drug sensitivity analysis. [file Image_4.tif]
